# Circulating memory PD-1^+^CD8^+^ T cells and PD-1^+^CD8^+^T/PD-1^+^CD4^+^T cell ratio predict response and outcome to immunotherapy in advanced gastric cancer patients

**DOI:** 10.1186/s12935-023-03137-9

**Published:** 2023-11-16

**Authors:** Jiang Liu, Degan Liu, Guangyin Hu, Jingjing Wang, Dadong Chen, Chuanjun Song, Yin Cai, Chentong Zhai, Wenjing Xu

**Affiliations:** Department of Oncology, The Affiliated Xinghua People’s Hospital, Medical School of Yangzhou University, 419 Ying Wu Nan Road, Xinghua, 225700 Jiangsu People’s Republic of China

**Keywords:** Gastric cancer, Immunotherapy, Immune checkpoint inhibitors, Memory CD8^+^ T cells, CD8^+^T/CD4^+^T cell ratio, Predictive biomarkers

## Abstract

**Background:**

Limited benefit population of immunotherapy makes it urgent to select effective biomarkers for screening appropriate treatment population. Herein, we have investigated the predictive values of circulating CD8^+^ T cells and CD8^+^T/CD4^+^T cell ratio in advanced gastric cancer patients receiving immunotherapy.

**Methods:**

A retrospective cohort analysis of 187 advanced gastric cancer patients receiving sintilimab combined with oxaliplatin and capecitabine therapy in The Affiliated Xinghua People’s Hospital, Medical School of Yangzhou University between December 2019 and February 2023 was conducted. The corresponding clinical outcomes of the variables were analyzed by receiver operating characteristic (ROC) curve, chi-square test, Kaplan–Meier methods and Cox proportional hazards regression models.

**Results:**

The optimal cutoff values for percentages of CD8^+^ T cells, naive CD8^+^ T cells (CD8^+^ Tn) and memory CD8^+^ T cells (CD8^+^ Tm) expressing programmed cell death -1(PD-1) as well as PD-1^+^CD8^+^T/PD-1^+^CD4^+^T cell ratio were 21.0, 21.5, 64.3 and 0.669, respectively. It was found that the mean percentages of CD8^+^ T and CD8^+^ Tm expressing PD-1 as well as PD-1^+^CD8^+^T/PD-1^+^CD4^+^T cell ratio were significantly higher in responder (R) than non-responder (NonR) advanced gastric cancer patients associated with a longer progression free survival (PFS) and overall survival (OS). We also observed this correlation in programmed cell death-ligand 1(PD-L1) combined positive score (CPS) ≥ 5 subgroups. Univariate and multivariate Cox regression analyses demonstrated that lower CD8^+^ T and CD8^+^ Tm expressing PD-1 as well as PD-1^+^CD8^+^T/PD-1^+^CD4^+^T cell ratio were independent risk factors in advanced gastric cancer patients receiving immunotherapy plus chemotherapy.

**Conclusion:**

The circulating memory PD-1^+^CD8^+^ T cells and PD-1^+^CD8^+^T/PD-1^+^CD4^+^T cell ratio revealed high predictive values for response and prolonged survival outcomes in advanced gastric cancer patients receiving immunotherapy. Memory PD-1^+^CD8^+^ T cells and PD-1^+^CD8^+^T/PD-1^+^CD4^+^T cell ratio might be effective for screening benefit population of immunotherapy in advanced gastric cancer patients based on this preliminary evidence.

## Introduction

Gastric cancer is one of the common causes of cancer-related death due to lack of effective therapies [1]. Despite the advance in multidisciplinary treatment, the prognosis of these patients remains poor [2]. The development of immunology has greatly promoted the progress of cancer immunotherapy. In the recent years, immunotherapy has become a novel treatment modality and its anti-tumor effect has been affirmed by plenty of clinical studies [3–6]. Some encouraging clinical studies of gastric cancer have established immune checkpoint inhibitors (ICI) therapies as one of the new treatment criteria for gastric cancer. The significant efficacy and sustained response of ICI therapies were demonstrated in the third-line treatment of gastric cancer in several studies [7, 8]. Moreover, the findings of two other studies [9, 10] further demonstrated that first-line ICI therapies combined with chemotherapy could significantly improve objective response rate (ORR) and prolong survival time in advanced gastric cancer patients, providing a consolidated basis for first-line ICI therapies of gastric cancer. In addition, KEYNOTE-811study [11] showed that first-line pembrolizumab combined with trastuzumab and chemotherapy dramatically reduces tumour size, induces complete responses and significantly improves ORR in some human epidermal growth factor receptor-2(Her2)-positive advanced gastric cancer patients. However, benefit population of ICI therapies in gastric cancer patients are very limited in the real world, implicating screening appropriate treatment population remains an urgent challenge to be solved. Some tissue biomarkers such as PD-L1, mismatch repair defects (MMR) and tumor mutation burden (TMB), have been often used to screen populations who benefit from ICI therapies [12–14]. There are therefore unmet clinical needs to select novel biomarkers for stratifying the patients.

PD-1 / PD-L1 antibodies are immune checkpoint blocking drugs. Its mechanism of action is that tumor cells bind to PD-1 of T cells through PD-L1, thus escaping the recognition of T cells. PD-1/PD-L1 antibodies block this process and restore the ability of T cells to recognize and kill tumor cells. The process includes tumor cell antigen release, tumor antigen presentation, initiation and activation, T cell transported to tumor tissue, T cell infiltrated to tumor tissue, and T cell recognizing and killing tumor cells. T cells are consumed in the process of killing tumor cells. Therefore, the distribution and number of T cell subsets will affect anti-tumor effect of PD-1/PD-L1 antibodies. In addition, the activity and number of memory T cells also play an important role in the anti-tumor therapy of immune checkpoint inhibitors. And any factor in this process would affect the therapeutic effect of immune checkpoint inhibitors.

The host immune system plays a key role in controlling and eliminating cancer [15]. Cytotoxic CD8^+^T cells have been considered the main effectors of anti-tumor immune responses. The correlations between CD8^+^ T cells and the responses to ICI therapies have been explored in some tumors. In a small cohort study of non-small-cell lung cancer patients, the presence of CD8^+^ T cells expressing high levels of PD-1 was strongly predictive for response and survival outcome to anti-PD-1 therapy [16]. Kamphorst et al. also discovered that proliferation of PD-1^+^ CD8^+^ T cells in peripheral blood after anti-PD-1 therapy is associated with good response and prognosis in lung cancer patients [17]. An American study revealed that exhausted TCF1^+^Tim-3^−^ CD8^+^ tumor-infiltrating lymphocytes (TILs) not exhausted TCF1^−^Tim-3^+^ CD8^+^ TILs can respond to anti-PD-1 therapy and may be an effective predictor of immunotherapy in melanoma patients [18]. In another study, higher percentages of TCF1-expressing PD-1-positive CD8^+^ T Cells are associated with the proliferative response to ICI therapy in melanoma patients [19]. Although further analysis of pre- and post-treatment tumor biopsy may be more valuable than blood analysis, there are limitations in the analysis of tumor sites, especially in visceral tumors patients. Tumor tissues are sometimes difficult to obtain or repeatedly obtain in clinical practice. Peripheral blood analyses are easier to perform, can be repeated dynamically, and may provide a new insight for immunotherapy. In view of the important role of CD8^+^ T cells in the immune response, we hypothesized that it may have some predictive values for the effect of ICI therapies. Therefore, in the present study, we evaluated significances of CD8 ^+^ T cells in predicting the responses to ICI therapies in advanced gastric cancer patients.

## Materials and methods

### Patients

**Fig. 1:**
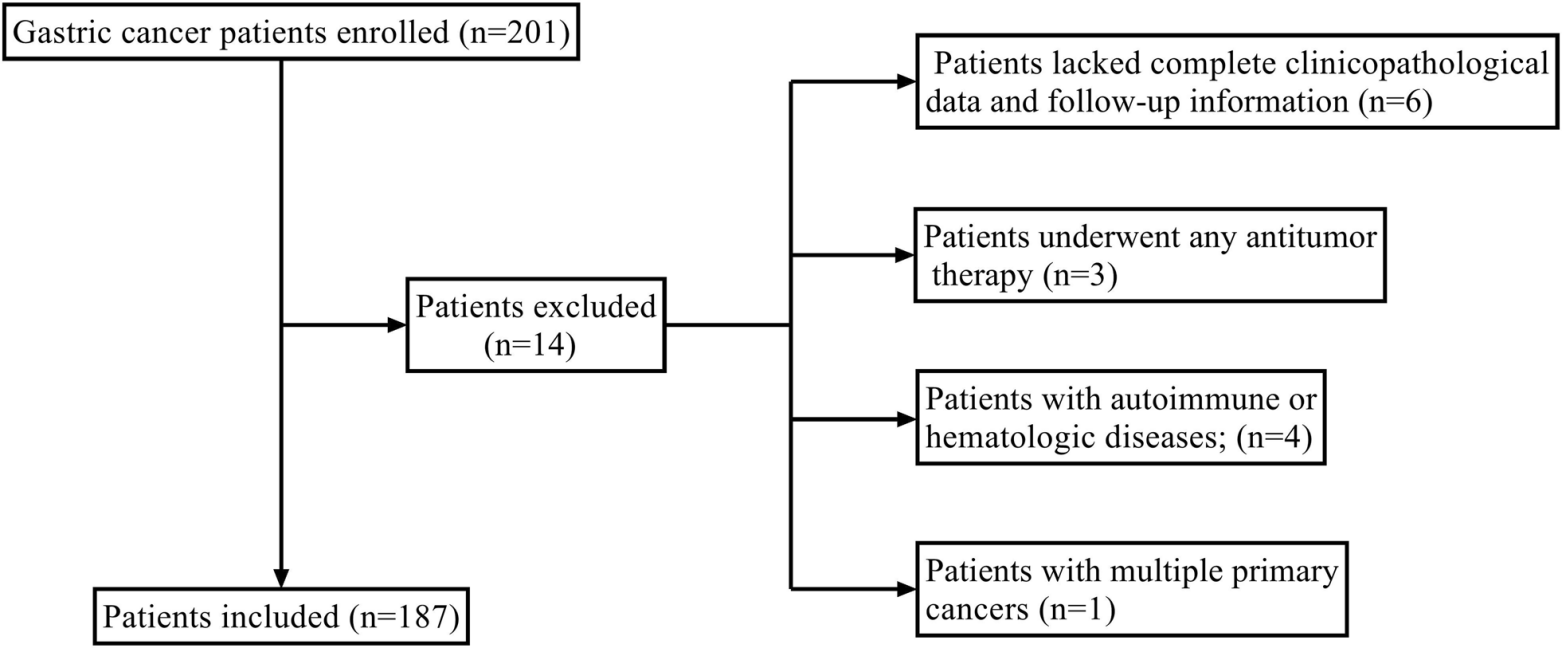
The flowchart of the enrollment process

A total of 187 advanced gastric cancer patients treated with sintilimab combined with capecitabine and oxaliplatin were enrolled in The Affiliated Xinghua People’s Hospital, Medical School of Yangzhou University in our study. All patients underwent 200 mg sintilimab plus 1000 mg/m^2^ (twice a day) capecitabine and 130 mg/m^2^ oxaliplatin every 3 weeks until disease progression or intolerable toxicity. The inclusion criteria were as follows: (i) patients over 18 years old; (ii) patients whose pathologic diagnosis was Gastric adenocarcinoma or adenocarcinoma of gastroesophageal junction; (iii) patients treated with at least 3 cycles of sintilimab plus chemotherapy; (iv) Eastern Cooperative Oncology Group Performance Status score at 0–2; (v) patients with complete evaluable imaging data and peripheral hematological parameters before treatment; and (vi) patients at clinical stage III-IV, according to the eighth edition of the American Joint Committee on Cancer staging manual. The exclusion criteria were as follows: (i) patients lacked complete clinicopathological data and follow-up information; (ii) patients underwent any antitumor therapy; (iii) patients with autoimmune or hematologic diseases; (iv) patients with multiple primary cancers. Fourteen patients were excluded and one hundred and eighty-seven patients were eventually enrolled. The whole enrollment process was displayed in Fig. 1. Our study involving human participants was reviewed and approved by the Ethics Committee of The Affiliated Xinghua People’s Hospital, Medical School of Yangzhou University. All subjects gave written informed consent in accordance with the Declaration of Helsinki.

### Evaluation of efficacy

Gastroscopy and low-dose computed tomography scan were performed before treatment and every 8 weeks after treatment. We assessed response and efficacy with Response Evaluation Criteria in Solid Tumors version 1.1 (RECIST 1.1) every 8 weeks [20]. Three radiologists were asked to evaluate response and efficacy for every patient. Patients were accordingly defined as R group with complete response (CR), partial response (PR) or stable disease (SD) with no progression over six months, and NonR group with progressive disease (PD) within 6 months [21]. OS time which was defined as the time from initial treatment to death from any cause was the primary endpoint in our study. For patients who had been lost to follow-up prior to death, the last follow-up time was regarded as the equivalent to time of death. PFS time, defined as the time from the start of treatment to disease progression or death from any cause, was the secondary endpoint.

### Blood cell analyses and flow cytometric analyses

Tubes containing ethylene diaminetetraacetic acid (EDTA) were used to collect peripheral venous blood between 6:00 am and 8:00 am. Peripheral blood mononuclear cells (PBMCs) were isolated immediately by density gradient centrifugation using LymphoprepTM reagent (Axis-shield, Norway) based on manufacturer's instructions.

All samples were divided and every 50 μL aliquots were incubated with the mixtures of different fluorochrome-conjugated antibodies in 100 μL FACS buffer (phosphate buffer saline (PBS) plus 2% fetal bovine serum (FBS)) (Millipore, USA) for 30 min at 4 °C in the dark. Cells were washed once using PBS and resuspended in PBS. Acquisition was performed on LSR Fortessa flow cytometry (BD Pharmingen). Data analyses were performed with FlowJo software (version 10). In order to ensure quality control and comparability of results, flow cytometry was corrected every day, and antibody fluorescence intensity was monitored weekly.

### Statistical analyses

Statistical analyses were performed using IBM SPSS Statistic 26.0 (IBM Corp.) and GraphPad Prism 9.3. Receiver operating characteristic (ROC) curves were constructed to determine optimal cut-off values for relevant variables. The χ^2^ test was used to analyze the associations between peripheral blood parameters and clinical characteristics of patients. The differences between R and NonR groups were compared by a Wilcoxon test. Survival analyses were performed using the Kaplan–Meier method (log-rank test). Univariate and multivariate analyses of relevant variables related to ICI therapy were tested by Cox proportional hazard models. All statistical analyses were two-sided probability tests (α = 0.05) and *P* < 0.05 was considered statistically significant.

## Results

### Patient characteristics

A total of 187 advanced gastric cancer patients receiving sintilimab plus capecitabine and oxaliplatin treatment were enrolled in this study. The median age was 62 years (range, 36–82 years) at the time of diagnosis. Male patients accounted for 77% of all participants. The proportion of patients with a drinking history was close to 75%. The proportion of poorly differentiated and well or moderately differentiated patients was 33.7 and 66.3%, respectively. More than 70% of participants were of clinical stage III. One hundred and twenty-two (65.2%) participants were classified as R group and sixty-five (34.8%) participants were defined as NonR group according to the RECIST 1.1 criteria. By February 2023, the median follow-up time was 12.6 months (range, 3.2–32.5 months).

### The distribution of T cell subsets in PD-1 positive lymphocytes

T cell subsets of lymphocytes expressing PD-1 in peripheral blood of 187 patients with advanced gastric cancer were performed by multiplex flow cytometry.

**Fig. 2:**
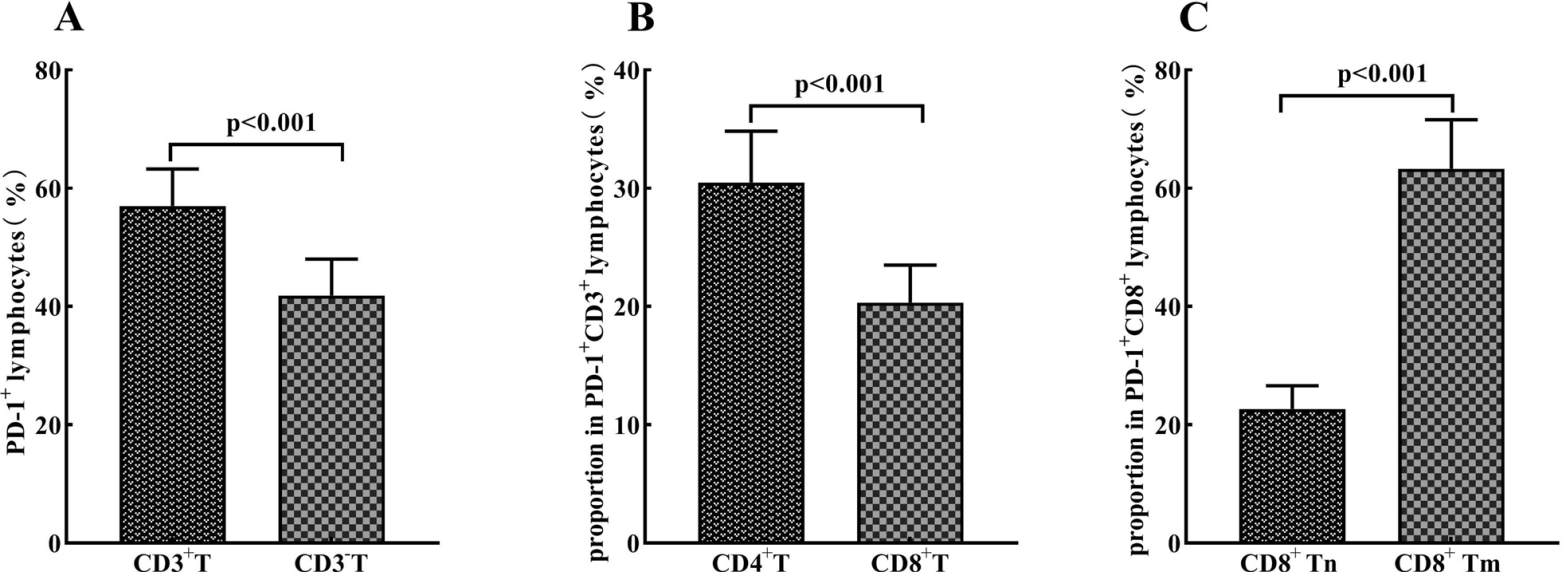
The distribution of T cell subsets in PD-1 positive lymphocytes in peripheral blood. The average percentages of CD3^+^ and CD3^−^ cells (**A**), CD4^+^ T cells and CD8^+^ T cells (**B**) in peripheral lymphocytes expressing PD-1 in advanced gastric cancer patients before receiving sintilimab plus capecitabine and oxaliplatin treatment. The average percentages of CD8^+^Tm and CD8^+^Tn in PD-1 positive CD8^+^ T cells in peripheral blood (**C**)

It was showed that the mean percentage of CD3^+^ T cells (56.98 ± 6.29%) in PD-1 positive lymphocytes was significantly higher than that of CD3^−^ T cells (41.88 ± 6.15%) (*P* < 0.001) (Fig. 2A). The mean percent of CD8^+^ T cells (20.33 ± 3.18%) was lower than that of CD4^+^ T cells (30.48 ± 4.34%) (*P* < 0.001) among.

PD-1^+^CD3^+^ T cells (Fig. 2B). The distribution of PD-1 ^+^CD8^+^ T cell subsets was also analyzed based on CD45RA and CD45RO expressions. It was revealed that PD-1 ^+^CD8^+^ Tm (CD45RA − CD45RO +) (63.28 ± 8.31%) were dominant subsets, whereas PD-1 ^+^CD8^+^ Tn (CD45RA + CD45RO −) (22.65 ± 3.96%) were less frequent (Fig. 2C). These results imply that peripheral PD-1^+^CD8^+^ Tm cells are the leading subset among CD8^+^ lymphocytes expressing PD-1 in peripheral blood.

### ***Determination of optimal cut-off values for PD-1***^+^***CD8***^+^***T cells subsets and PD-1***^+^***CD8***^+^***T/PD-1***^+^***CD4***^+^***T cell ratio***

**Fig. 3:**
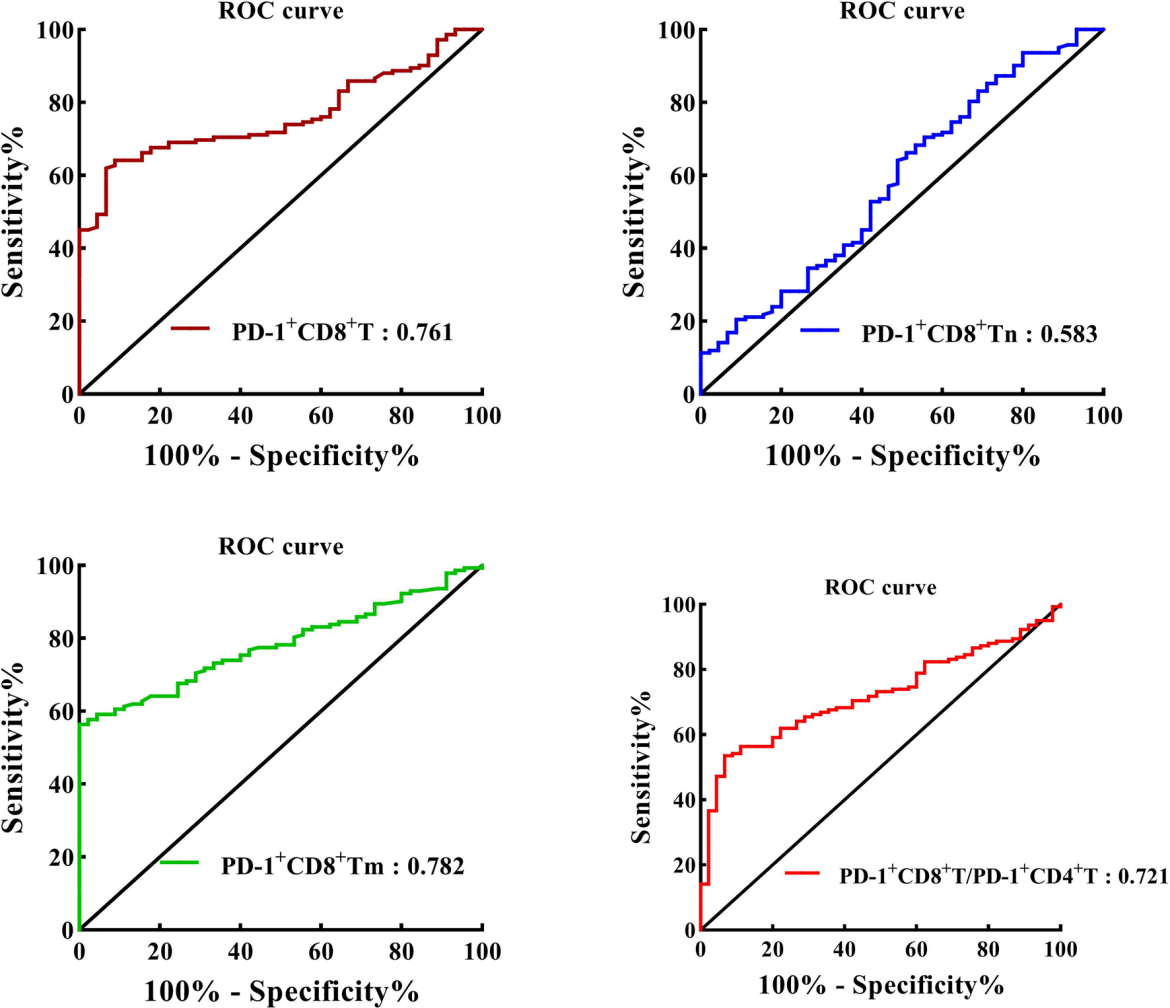
ROC curves analyses for the optimal cut-off values of CD8^+^ T, CD8^+^ Tn and CD8^+^ Tm expressing PD-1 as well as PD-1^+^CD8^+^T/PD-1^+^CD4^+^T cell ratio, respectively. The areas under the ROC curves of CD8^+^ T, CD8^+^ Tn and CD8^+^ Tm expressing PD-1 as well as PD-1^+^CD8^+^T/PD-1^+^CD4^+^T cell ratio are indicated

As revealed in Fig. 3, the areas under ROC curves for percentages of PD-1^+^CD8^+^ T in peripheral PD-1^+^CD3^+^ T cells, PD-1^+^CD8^+^ Tn and PD-1^+^ CD8^+^ Tm in peripheral PD-1^+^ CD8^+^ T cells as well as PD-1^+^CD8^+^T/PD-1^+^CD4^+^T cell ratio were 0.761, 0.583, 0.782 and 0.721, respectively. The optimal cut-off values for percentages of PD-1^+^CD8^+^ T, PD-1^+^CD8^+^ Tn and PD-1^+^ CD8^+^ Tm as well as PD-1^+^CD8^+^T/PD-1^+^CD4^+^T cell ratio were 21.0, 21.5, 64.3% and 0.669, respectively. Patients were separately divided into high and low groups based on the optimal cut-off values.

### Associations between clinicopathological characteristics of patients and peripheral blood parameters

The associations between clinicopathological characteristics of patients and peripheral blood relevant parameters were displayed in Table 1. The percentages of PD-1^+^CD8^+^ T and PD-1^+^CD8^+^ Tm as well as PD-1^+^CD8^+^T/PD-1^+^CD4^+^T cell ratio prior to treatment were significantly associated with response and PD-L1 CPS (*P* < 0.001). There were slightly statistical differences between PD-1^+^CD8^+^T/PD-1^+^CD4^+^T cell ratio before treatment and clinical stage and differentiation (*P* = 0.017 and *P* = 0.046).

**Table 1:** Associations between peripheral blood parameters and clinical characteristics of advanced gastric cancer patients

Variable	Cases, n	PD-1^+^CD8^+^T (%)			PD-1^+^CD8^+^Tm (%)			PD-1^+^CD8^+^T/PD-1^+^CD4^+^T ratio		
		≥ 21.0, n	< 21.0, n	P-value	≥ 64.3, n	< 64.3, n	P-value	≥ 0.669, n	< 0.669, n	P-value
Total patients	187	90	97		81	106		79	108	
Age, years				0.355			0.177			0.266
≥ 60	112	57	55		53	59		51	61	
< 60	75	33	42		28	47		28	47	
Sex				0.916			0.896			0.953
Male	144	69	75		62	82		61	83	
Female	43	21	22		19	24		18	25	
Drinking history				0.332			0.654			0.967
Yes	137	63	74		58	79		58	79	
No	50	27	23		23	27		21	29	
ECOG PS				0.093			0.068			0.058
0–1	113	60	53		55	58		54	59	
2	74	30	44		26	48		25	49	
Stage				0.495			0.376			0.017
III	137	68	69		62	75		65	72	
IV	50	22	28		19	31		14	36	
Differentiation				0.099			0.475			0.046
Well or moderate	124	65	59		56	68		46	78	
Poor	63	25	38		25	38		33	30	
Response				< 0.001			< 0.001			< 0.001
R	122	90	32		80	42		70	52	
NonR	65	0	65		1	64		9	56	
PD-L1 CPS				< 0.001			< 0.001			< 0.001
≥ 5	104	81	23		78	26		65	39	
< 5	83	9	74		3	80		14	69	

*PD-1*^*+*^*CD8*^*+*^*T* PD-1^+^CD8^+^ T cells, *PD-1*^*+*^*CD8*^*+*^*Tm* memory PD-1^+^CD8^+^ T cells, *PD-1*^*+*^*CD4*^*+*^*T* PD-1^+^CD4^+^ T cells, *CPS* combined positive score, *ECOG PS* Eastern Cooperative Oncology Group Performance Status

### Impacts of peripheral blood relevant parameters on survival outcome and response to ICI therapy

**Fig. 4:**
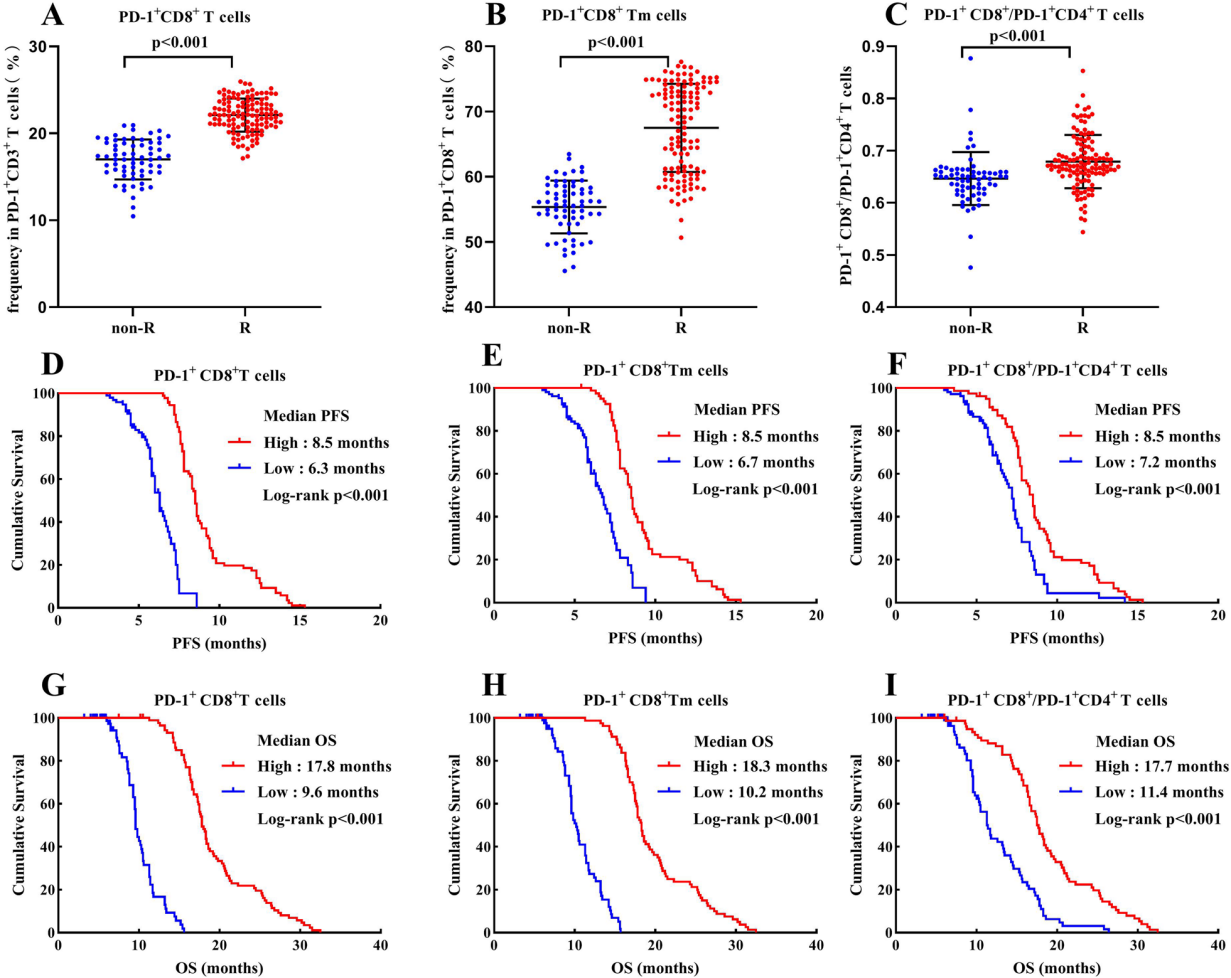
**A**–**C**: Comparison of the percentages of PD-1^+^ CD8^+^ T cells (**A**), PD-1^+^ CD8^+^ Tm cells (**B**) and PD-1^+^CD8^+^T/PD-1^+^CD4^+^T cell ratio (**C**) between R and NonR advanced gastric cancer patients. **D**–**I**: Kaplan Meier curves illustrating the impact of PD-1^+^ CD8^+^ T cells (**D**, **G**), PD-1^+^ CD8^+^ Tm cells (**E**, **H**) and PD-1^+^CD8^+^T/PD-1^+^CD4^+^T cell ratio (**F**, **I**) on PFS and OS, respectively

**Fig. 5:**
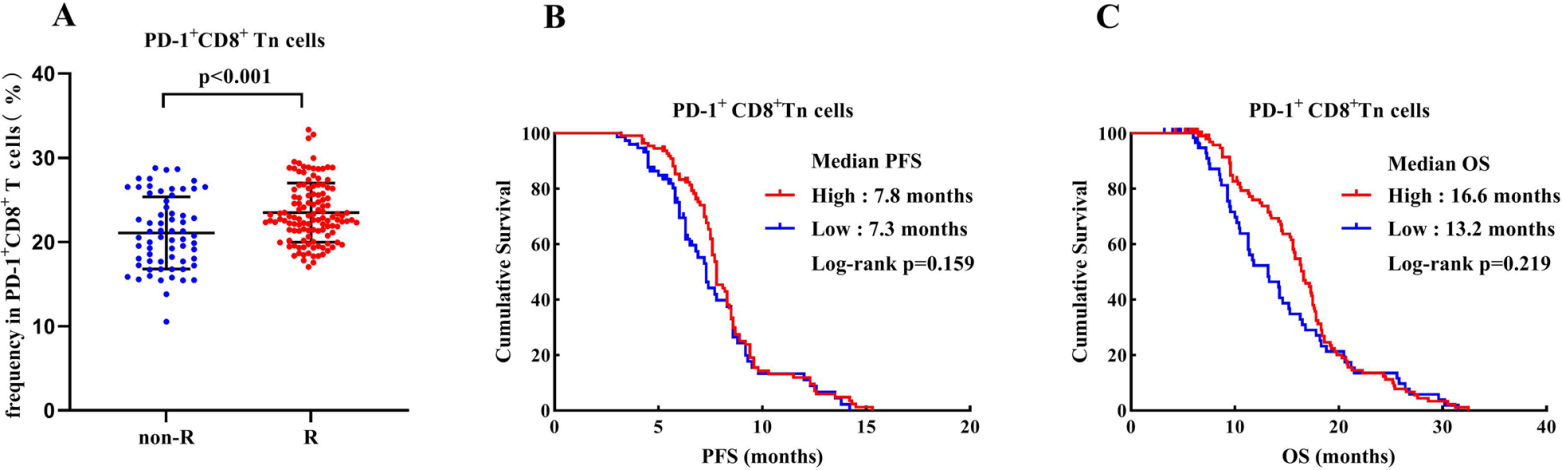
**A**: Comparison of the percentages of PD-1^+^ CD8^+^ Tn cells between R and NonR advanced gastric cancer patients. **B**, **C**: Kaplan Meier curves illustrating the impact of PD-1^+^ CD8^+^ Tn cells on PFS and OS

To explore the associations of CD8^+^ T cells in the peripheral blood with the responses to ICI therapy in patients with advanced gastric cancer, some CD8^+^ T cell subsets before receiving sintilimab plus oxaliplatin and capecitabine therapy were firstly determined and compared between R and NonR gastric cancer patients. Our results revealed that the average percentages of PD-1^+^CD8^+^ T in peripheral PD-1^+^CD3^+^ T cells, PD-1^+^CD8^+^ Tn and PD-1^+^ CD8^+^ Tm in peripheral PD-1^+^ CD8^+^ T cells as well as PD-1^+^CD8^+^T/PD-1^+^CD4^+^T cell ratio were significantly higher in R groups than that in NonR groups (Figs. 4A–C, 5A). Patients with high percentages of PD-1^+^CD8^+^ T (median PFS: high vs. low = 8.5 vs. 6.3 months, *P* < 0.001; median OS: 17.8 vs. 9.6 months, *P* < 0.001; Fig. 4D, G) and PD-1^+^CD8^+^ Tm (median PFS: high vs. low = 8.5 vs. 6.7 months, *P* < 0.001; median OS: 18.3 vs. 10.2 months, *P* < 0.001; Fig. 4E, H) as well as PD-1^+^CD8^+^T/PD-1^+^CD4^+^T cell ratio (median PFS: high vs. low = 8.5 vs. 7.2 months, *P* < 0.001; median OS: 17.7 vs. 11.4 months, *P* < 0.001; Fig. 4F, I) at the baseline indicated a good prognosis with significantly prolonged PFS and OS. However, there was no statistical difference between the percentage of PD-1^+^CD8^+^Tn (median PFS: high vs. low = 7.8 vs. 7.3 months, *P* = 0.159; median OS: 16.6 vs. 13.2 months, *P* = 0.219; Fig. 5B, C) and survival times.

### *Peripheral blood relevant parameters in predicting the response and survival outcome to ICI therapy in CPS* ≥ *5 populations*

**Fig. 6:**
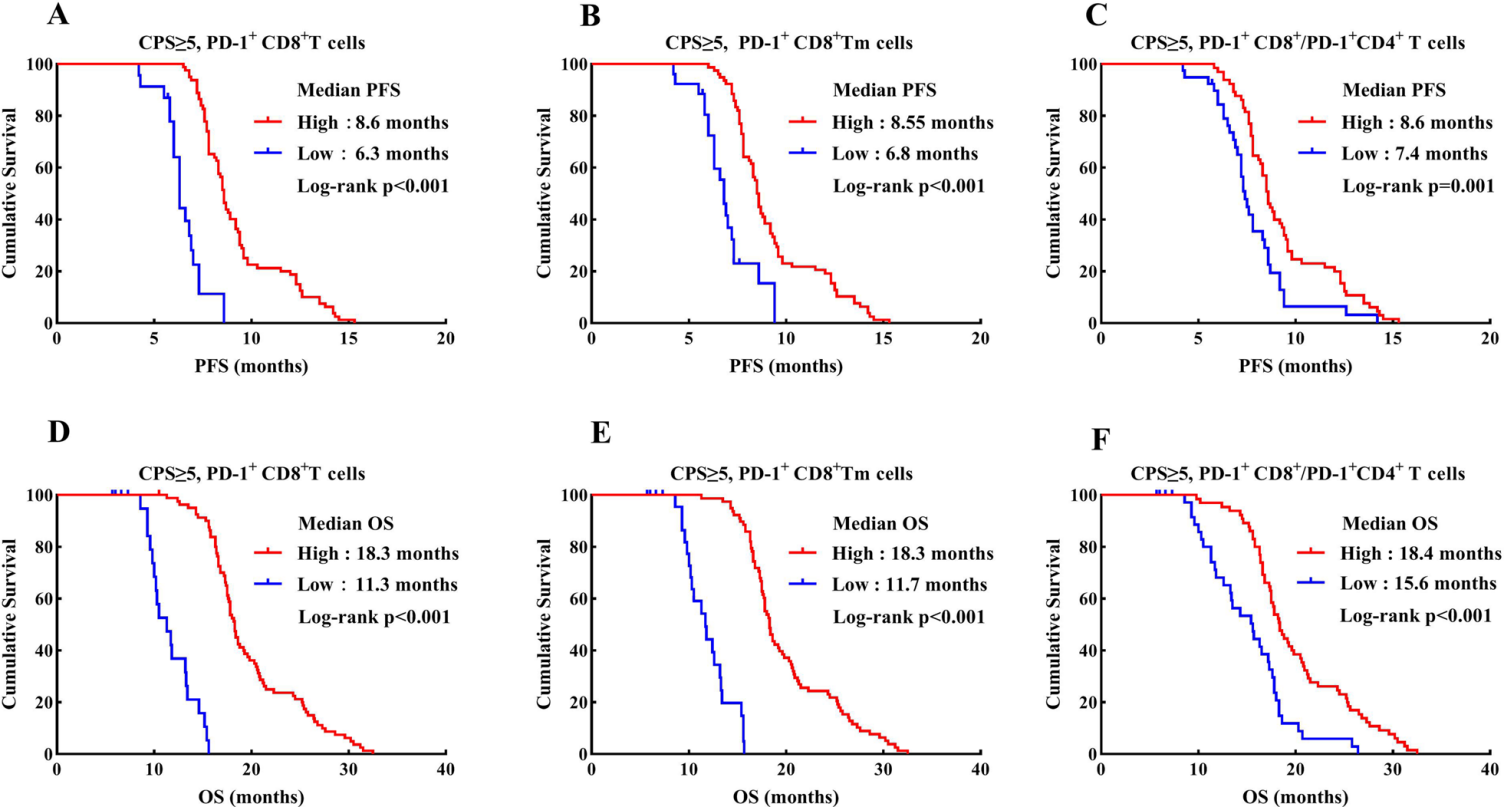
Kaplan Meier curves demonstrating the impact of PD-1^+^ CD8^+^ T cells (**A**, **D**), PD-1^+^ CD8^+^ Tm cells (**B**, **E**) and PD-1^+^CD8^+^T/PD-1^+^CD4^+^T cell ratio (**C**, **F**) on PFS and OS in the PD-L1 CPS ≥ 5 subgroups, respectively

We further investigated the predictive values of peripheral CD8^+^ T cell subsets in CPS ≥ 5 subgroups. Except for PD-1^+^CD8^+^ Tn cells, the percentages of PD-1^+^CD8^+^ T and PD-1^+^CD8^+^ Tm as well as PD-1^+^CD8^+^T/PD-1^+^CD4^+^T cell ratio prior to treatment had a significant effect on survival outcomes in our results. Patients in CPS ≥ 5 subgroups with high frequencies of PD-1^+^CD8^+^ T (median PFS: high vs. low = 8.6 vs. 6.3 months, *P* < 0.001; median OS: 18.3 vs.11.3 months, *P* < 0.001; Fig. 6A, D) and PD-1^+^CD8^+^ Tm (median PFS: high vs. low = 8.55 vs. 6.8 months, *P* < 0.001; median OS: 18.3 vs. 11.7 months, *P* < 0.001; Fig. 6B, E), as well as high PD-1^+^CD8^+^T/PD-1^+^CD4^+^T cell ratio (median PFS: high vs. low = 8.6 vs. 7.4 months, *P* = 0.001; median OS: 18.4 vs. 15.6 months, *P* < 0.001; Fig. 6C, F) at the baseline also displayed significantly prolonged survival times.

### Prognostic value of peripheral blood relevant parameters in ICI therapy of advanced gastric cancer patients

The prognostic values of PD-1^+^ CD8^+^ T cells subsets were further assessed by Cox proportional hazards models. The results of the univariate and multivariate analyses of PFS and OS were showed in Tables 2, 3, respectively. Univariate analyses presented that high PD-L1 CPS, percentages of PD-1^+^CD8^+^ T and PD-1^+^CD8^+^ Tm, as well as high PD-1^+^CD8^+^T/PD-1^+^CD4^+^T cell ratio groups before treatment were associated with prolonged PFS and OS times (all *P* < 0.001). In the multivariate analyses, to avoid the multicollinearity among T cells subsets, five independent Cox models were separately constructed. Each model included only one parameter. The multivariate analyses indicated that PD-L1 CPS, percentages of PD-1^+^CD8^+^ T and PD-1^+^CD8^+^ Tm, as well as PD-1^+^CD8^+^T/PD-1^+^CD4^+^T cell ratio were independent prognostic factors (all *P* < 0.05) for survival times in advanced gastric cancer patients receiving sintilimab plus capecitabine and oxaliplatin treatment.

**Table 2:** Univariate and multivariate analyses of progression-free survival in advanced gastric cancer patients receiving immunotherapy and chemotherapy

Variable	Univariate analysis		Multivariate analysis	
	HR (95% CI)	P-value	HR (95% CI)	P-value
Age (≥ 60vs. < 60 years)	1.210 (0.856–1.712)	0.280		
Sex (male vs. female)	0.802 (0.538–1.195)	0.278		
Drinking history (yes vs. no)	0.986 (0.678–1.435)	0.942		
ECOG PS (0–1 vs. 2)	0.798 (0.566–1.124)	0.197		
Stage (III vs. IV)	0.723 (0.497–1.052)	0.090		
Differentiation (well or moderate vs. poor)	1.157 (0.806–1.662)	0.430		
PD-1^+^CD8^+^Tn % (< 21.5 vs. ≥ 21.5)	1.273(0.901–1.797)	0.171		
PD-L1 CPS (< 5 vs. ≥ 5)	3.538(2.297–5.450)	< 0.001	3.052 (1.909–4.879)	< 0.001
PD-1^+^CD8^+^T % (< 21.0 vs. ≥ 21.0)	9.178(5.592–15.06)	< 0.001	8.124 (4.532–14.56)	< 0.001
PD-1^+^CD8^+^Tm % (< 64.3 vs. ≥ 64.3)	4.096 (2.740–6.121)	< 0.001	3.551 (2.077–6.070)	< 0.001
PD-1^+^CD8^+^T/PD-1^+^CD4^+^T ratio(< 0.669 vs. 0.669)	2.137 (1.500–3.043)	< 0.001	1.780 (1.178–2.690)	0.006

*PD-1*^*+*^*CD8*^*+*^*T* PD-1^+^CD8^+^ T cells, *PD-1*^*+*^*CD8*^*+*^*Tm* memory PD-1^+^CD8^+^ T cells, *PD-1*^*+*^*CD4*^*+*^*T* PD-1^+^CD4^+^ T cells, *CPS* combined positive score, *ECOG PS* Eastern Cooperative Oncology Group Performance Status

**Table 3:** Univariate and multivariate analyses of overall survival in advanced gastric cancer patients receiving immunotherapy and chemotherapy

Variable	Univariate analysis		Multivariate analysis	
	HR (95% CI)	P-value	HR (95% CI)	P-value
Age (≥ 60vs. < 60 years)	1.144 (0.808–1.620)	0.447		
Sex (male vs. female)	0.799 (0.535–1.195)	0.274		
Drinking history (yes vs. no)	1.016 (0.700–1.476)	0.932		
ECOG PS (0–1 vs. 2)	0.762 (0.540–1.074)	0.120		
Stage (III vs. IV)	0.739 (0.509–1.075)	0.113		
Differentiation (well or moderate vs. poor)	1.169 (0.815–1.678)	0.396		
PD-1^+^CD8^+^Tn % (< 21.5 vs. ≥ 21.5)	1.239(0.878–1.748)	0.223		
PD-L1 CPS (< 5 vs. ≥ 5)	11.86(6.934–20.28)	< 0.001	5.224 (2.802–9.740)	< 0.001
PD-1^+^CD8^+^T % (< 21.0 vs. ≥ 21.0)	23.12 (12.63–42.34)	< 0.001	16.92 (8.505–33.65)	< 0.001
PD-1^+^CD8^+^Tm % (< 64.3 vs. ≥ 64.3)	28.93 (14.48–57.77)	< 0.001	22.82 (10.39–50.14)	< 0.001
PD-1^+^CD8^+^T/PD-1^+^CD4^+^T ratio(< 0.669 vs. 0.669)	2.966 (2.071–4.248)	< 0.001	2.640(1.734–4.019)	< 0.001

*PD-1*^*+*^*CD8*^*+*^*T* PD-1^+^CD8^+^ T cells, *PD-1*^*+*^*CD8*^*+*^*Tm* memory PD-1^+^CD8^+^ T cells, *PD-1*^*+*^*CD4*^*+*^*T* PD-1^+^CD4^+^ T cells, *CPS* combined positive score, *ECOG PS* Eastern Cooperative Oncology Group Performance Status

## Discussion

Gastric cancer is a global health problem, with heavy social economic burden, high incidence and mortality [22]. Previously, the main treatments such as surgery, chemoradiotherapy and molecular targeted therapy failed to significantly prolong survival times of advanced gastric cancer patients [23]. ICI therapies, as novel treatments, bring hope to advanced gastric cancer patients. Two three-phase randomized controlled studies demonstrated that whether the PD-L1 CPS was more than five or not, ICI therapy plus chemotherapy compared with chemotherapy as first-line treatment significantly improved survival times in patients with advanced gastric or gastro-oesophagealjunction (GC/GEJ) adenocarcinoma cancer [9, 10]. However, benefit population of ICI therapies is very limited. Therefore, there is an urgent clinical need to explore biomarkers for predicting response and resistance to ICI therapies. In our study, we investigated the potential values of circulating CD8^+^ T cell subsets in predicting responses and effects to ICI therapy plus chemotherapy in Chinese patients with advanced gastric cancer. We discovered that the mean percentages of PD-1^+^CD8^+^ T, PD-1^+^CD8^+^ Tn and PD-1^+^CD8^+^ Tm as well as PD-1^+^CD8^+^T/PD-1^+^CD4^+^T cell ratio in R groups were significantly higher than that in NonR groups. The high percentages of circulating PD-1^+^CD8^+^ T and PD-1^+^CD8^+^ Tm as well as PD-1^+^CD8^+^T/PD-1^+^CD4^+^T cell ratio at the baseline were significantly associated with good prognoses in advanced gastric cancer patients treated with ICI therapy plus chemotherapy.

In the antitumor immunity dominated by cellular immunity, T lymphocyte subsets of different functions protect the host against tumor by several mechanisms [24]. CD8^+^T cells are considered a major population of immune cells controlling and eliminating cancer cells. Cytotoxic CD8^+^ T cells eliminate tumor cells through several mechanisms, including secretion of Bgranule-associated enzymes (perforin and granzymes) and cytokines (γ-interferon and tumor necrosis factor), as well as the apoptosis of tumor cells initiated by the binding of FASL molecules to FAS molecules on tumor cells. In chronically infected mice, a CD8 ^+^ T cells subset with specific phenotype and gene expression program possesses stem cell-like characteristics and proliferates after blockade of PD-1 pathway [25]. A small sample study of melanoma patients demonstrated that PD-1^+^ CD8^+^ tumor-infiltrating lymphocytes (TILs) in the fresh tumor tissues were identified as tumor-reactive immune cells and implied that PD-1^+^ CD8^+^ TILs could function as a potential predictive biomarker of anti-tumor immunotherapy [26]. Another study in Singapore also validated that a high proportion of PD-1^+^CD8^+^ TILs in gastric cancer predicted better survival times and molecular characteristics of PD-1 positive CD8 ^+^ T cells was associated with CD8 cytolytic activity, proliferation and activation [27]. Besides, Mazzaschi G et al. prospectively analyzed the PD-1^+^CD8^+^ T levels of peripheral blood in NSCLC patients receiving ICI therapies as first or more line treatment, and discovered that high PD-1^+^CD8^+^ T had positive impact on response and survival times [28]. However, the prognostic significance of PD-1 positive CD8^+^T-cells in gastric cancer remains controversial. Two findings [29, 30] demonstrated that circulating and intratumoral PD1^+^CD8^+^ T-cells are related to worse response and survival outcomes, and serve as an independent adverse prognostic indicator in gastric cancer, which are contrary to the results of the other two studies [31, 32]. Mechanistically, blockade of PD-1/PD-L1 interactions can to a certain extent restore the functions of exhausted T cells with high expression of PD-1, resulting in enhanced anti-tumor immunity [33, 34]. In the present study, the distribution of PD-1 ^+^CD8^+^ T cell subsets based on CD45RA and CD45RO expressions was analyzed. We discovered that the mean percentages of CD8^+^ T, CD8^+^ Tn and CD8^+^ Tm expressing PD-1 were significantly higher in R than NonR patients before receiving ICI therapy. Furthermore, whether the PD-L1 CPS was more than five or not, CD8^+^ T and CD8^+^ Tm expressing PD-1, Except PD-1^+^CD8^+^ Tn, were also significantly associated with better response and survival outcomes in advanced gastric cancer patients treated with immunotherapy plus chemotherapy. These results preliminary imply the significance of CD8^+^Tm cells in ICI therapy.

Nevertheless, the impact of the balance between CD4^+^ T and CD8^+^ T in peripheral blood of cancer patients on ICI therapies is still unclear. In endometrial carcinoma, the CD4^+^/CD8^+^ TILs ratio has been reported to be significantly associated with expression of immune checkpoint proteins such as cytotoxic T-lymphocyte associated protein 4(CTLA-4) and PD-L1 [35]. The pre-treatment CD4^+^/CD8^+^ TILs ratio by immunohistochemistry on gastric cancer tissues revealed a remarkable correlation with lymphovascular invasion, TNM stage and response to neoadjuvant chemotherapy, and lower CD4^+^/CD8^+^ TILs ratio predicted significantly better survival outcomes [36]. The elevated circulating CD8^+^PD-1^+^/CD4^+^PD-1^+^ ratio prior to immunotherapy has also been shown to be correlated with good survival times in advanced non-small cell lung cancer patients treated with anti-PD-(L)1treatment [37]. Our findings revealed that the PD-1^+^CD8^+^T/PD-1^+^CD4^+^T ratio before treatment were significantly correlated with response and longer survival times, which was consistent with the above studies. However, Hernberg et al. discovered that the constantly increasing CD4^+^T/CD8^+^T ratio predicts good objective response and survival times in metastatic melanoma patients treated with Immunotherapy plus chemotherapy [38]. The above results suggest that the predictive value of the balance between CD4^+^ T and CD8^+^ T for tumor immunotherapy is still worth exploring.

To the best of our knowledge, the correlation between CD8^+^ T cell subsets and response and prognosis of advanced gastric cancer patients treated with ICI therapies remains unknown. This correlation was first demonstrated in our study. Peripheral blood parameters used in this study are easier to repeat dynamically and cost-effective in clinical practice. Limitations of our study have to be acknowledged. Firstly, due to retrospective monocentric design and relatively small sample analysis, it is difficult to exclude selection bias. Secondly, we failed to explore the correlation between TILs in tumor tissue and lymphocytes in peripheral blood. Finally, our study lacks analysis of their functional capacity and their tumor specificity.

## Conclusions

In summary, our study preliminarily presented relatively high value of peripheral memory PD-1^+^CD8 ^+^ T and PD-1^+^CD8^+^T/PD-1^+^CD4^+^T ratio in predicting response and prognosis to immunotherapy plus chemotherapy in patients with advanced gastric cancer.

## Data Availability

All data generated during this study are included in this article. Further enquiries can be directed to the corresponding authors.
